# Muscle weakness assessment in non-hypoxemic COPD out-patients at tertiary care hospitals

**DOI:** 10.12669/pjms.37.2.3127

**Published:** 2021

**Authors:** Faisal Faiyaz Zuberi, Bader Faiyaz Zuberi, Faiza Sadaqat Ali, Nimrah Bader

**Affiliations:** 1 Faisal Faiyaz Zuberi, Ojha Institute of Chest Diseases, Dow University of Health Sciences, Karachi, Pakistan; 2 Bader Faiyaz Zuberi, Department of Medicine, Dow Medical College, Dow University of Health Sciences, Karachi, Pakistan; 3 Faiza Sadaqat Ali, Department of Medicine, Dow Medical College, Dow University of Health Sciences, Karachi, Pakistan; 4 Nimrah Bader, Department of Internal Medicine, University of Oklahoma Health Sciences Center, Oklahoma City, OK, USA

**Keywords:** Sarcopenia, Hand grip strength, Chair to stand time, BMI, FEV_1_

## Abstract

**Objective:**

To determine frequency of Muscle Weakness in Non-Hypoxemic COPD out-patients and Comparison with age matched non-COPD Controls.

**Methods:**

This cross-sectional study was conducted at OPD of Ojha Institute of Chest Diseases and Medicine, Dow University of Health Sciences, Karachi, Pakistan, during the period 8^th^ September 2019 till 30^th^ May 2020. Patients of both genders aged 25-70 years who were satisfying GOLD criteria for COPD and having SpO_2_ ≥ 94% were included. An age matched control group was added as control. Hand Grip Strength (HGS) and Chair to Stand time (CST) were recorded.

**Results:**

Two hundred fifty-six patients were inducted with aged and BMI matched group of non-COPD Control patients in ratio of 1:2 (n = 128). Comparison of HGS between Control and COPD Groups showed significant weakness in COPD group. Significant weakness in lower limbs in COPD Group with longer timings to complete the task. Mean FEV_1_ had significant low values in COPD Group. Age correlated negatively with HGS & positively with CST. BMI correlated positively with FEV_1_ and CST but negatively with HGS. HGS correlated positively with FEV_1_ and no correlation was found with CST. No correlation was found of CST with FEV_1_.

**Conclusion:**

Muscle weakness in COPD patients was shown by simple validated bedside tools. The older COPD patients had less HGS and were slower in doing CST whereas those COPD ones who had higher FEV_1_ had more HGS.

**Abbreviations**
COPD: Chronic Obstructive Pulmonary Disease
BMI: Body Mass Index
FEV: Forced Expiratory Volume in first second
GOLD: Global Initiative for Chronic Obstructive Lung Disease
HGS: Hand Grip Strength
CST: Chair to Stand Time

## INTRODUCTION

Chronic Obstructive Pulmonary Disease (COPD) is affecting around 10% of population of over the age 40 years and 4% in general population.^1^ Currently it is the fourth common cause of death in world and by 2030 it would become the third most common reason for mortality.^2^ The Global Initiative for Chronic Obstructive Lung Disease (GOLD)^3^ defines COPD as “a common preventable and treatable disease characterized by persistent airflow limitation that is usually progressive and associated with an enhanced chronic inflammatory response in the airways and lungs to noxious particles and gases”.^3^ Among other factors, presence of sarcopenia is one of the factors for increased morbidity and mortality in COPD.^4^

Sarcopenia is related to increase further more complications as of osteopenia and related bone disease in COPD patients.^5^ Decreased muscle mass leads to muscle weakness that further deteriorate the quality of life and mobility in these patients. The impact of muscle weakness in COPD is not well documented in epidemiological studies and the exact nature of their relationship is still unclear. Decreased mobility leads to sedentary lifestyle and aggravates obesity which further leads to increase in complications including metabolic syndrome.^6^ Decrease in lean body mass is one of the factors and has been shown to be a reliable predictor of mortality in COPD.^7^ There is gradual decrease in muscle mass in COPD over the age of 50 years at the rate of 1-2% per year leading to frailty syndrome.^7^ This has been documented to occur in 20-40% of all COPD patients including 10-15% of those with normal weight.^8^ Decrease in exercise capacity in COPD limits the daily living physical activities thus increasing risk of exacerbations.^8^ Muscle strength is important factor determining physical activities and exercise capacity.

The current study was planned to document decrease in muscle strength in COPD in comparison with age matched non-controls in our population. This study is first time documentation of comparison of muscle strength in COPD from our area and will help to identify patients at risk thereby leading to better understanding and management of COPD. This study was conducted to determine frequency of muscle weakness in non-hypoxemic out-patients with COPD and comparison with age matched non-COPD controls

## METHODS

### Operational Definitions:

### COPD:

Diagnosed with spirometry as per GOLD guideline of post-bronchodilator FEV_1_/FVC <0.70.^9^

### Non-hypoxemia

Diagnosed if SpO_2_ is ≥ 94% on finger pulse oximetry.

### Hand Grip Testing (HGS)

Done using standard hand grip strength testing dynamometer (CAMRY, South El Monte, CA, USA). Patients were asked to squeeze the device by dominant hand with full force/strength that he/she can apply and hold for 03 seconds and reading on displayed were recorded in proforma. The procedure was repeated after one minute for two more times and highest value achieved was taken for analysis.

### Chair to Stand Time (CST)

Patients were asked to stand from chair and sit continuously without pause for five times. Time was measured in seconds using standard stopwatch.^10^

### Body Mass Index (BMI)

Calculated by formula BMI = weight (kg) / height (m)^2^. Asian BMI classification was used in this study. < 18.5 for underweight, 18.5-22.9 for normal-weight, 23.0-27.5 for overweight, and > 27.5 for obese.^11^

### GOLD Classification of airflow limitation severity in COPD:^9^

(based on post-bronchodilator FEV_1_)

GOLD 1—Mild: FEV_1_ ≥ 80% predicted

GOLD 2—Moderate: 50% ≤ FEV_1_ < 80% predicted

GOLD 3—Severe: 30% ≤ FEV_1_ < 50% predicted

GOLD 4—Very Severe: FEV_1_ < 30% predicted

This cross-sectional study was conducted in out-patient department (OPD) of Ojha Institute of Chest Diseases and Medical OPD of Civil Hospital Karachi from 8^th^ September 2019 till 30^th^ May 2020.Using reported frequency of sarcopenia in COPD of 14.5%,^12^ power of 95% and alpha of 0.01; sample size was calculated as 205. Sample size calculation was done by PASS 2019 software.

Convenience sampling technique was used. Study has ethical approval from IRB of Dow University of Health Sciences vide its letter # IRB-1419/DUHS/Approval/2019 dated 04 November 2019. Patients were informed about study protocol and possible adverse effects and given option to withdraw from study any time.

### Inclusion Criteria

Out-patients of both genders of age 25-70 years were included after informed consent. Patients presenting with symptoms suggestive of airway disease were evaluated in OPD for COPD. COPD was diagnosed as per operational criteria.

### Exclusion Criteria

Patients with finger pulse oximetry SpO_2_ of < 94% were excluded. Patients with pulmonary tuberculosis, pleural effusion, heart failure, diagnosed cases of myopathy, polymyositis, arthritis, diabetes were also excluded.

Finger pulse oximetry was done of all patients satisfying COPD criteria and those with oxygen saturations of ≥ 94% were included in study for further workup after informed consent. Demographics like age, gender, height and weight were recorded. Body mass index (BMI) was calculated. Hand Grip Strength (HGS) was measured in kilograms, three reading were taken one minute apart. Best of three was selected for analysis. Five-times Chair to Stand (CST) time was measured in seconds. An age matched control group was added as control in a ratio of 1:2. Post bronchodilatation FEV_1_ was recorded using Vitalograph spirometry machine.

### Analysis

Age, BMI, HGS, CST & FEV_1_ in both groups was compared by Student’s t-test. Intra-group comparison of age, HGS, CST & FEV_1_ on basis of gender was done using Student’s t-test. BMI values were segregated into BMI Groups based on its classification and their frequencies were compared between two groups using χ^2^ test. FEV_1_ values were segregated into groups as defined by GOLD Classification and were compared between groups using χ^2^ test. Significance level was set at ≤ 0.05. SPSS version 25 was used for statistical analysis.

## RESULTS

Two hundred fifty-six out-patients satisfying the selection criteria were inducted after informed consent were included in the study. An aged matched group of non-COPD Control patients in ratio of 1:2 also inducted as control (n = 128). There was no significant difference between age of two groups and were adequately matched (*t* (213.8) = -1.7, *p* = .084). The difference in BMI in both groups was statistically insignificant and both groups were adequately matched (t (336.6) = -1.86, p = .064). Comparison of Mean FEV_1_ in both groups with Student’s t-test showed its significant low values in COPD Group (t (382) = 27.3, p = < .001). Upper limb strength was measured with HGS and in comparison, of HGS between Control and COPD Groups showed significant weakness in COPD group (t (150.8) = 6.1, p < .001). Lower limb strength was measured with CST and in comparison, with Student’s t-test showed significant weakness in lower limbs in COPD Group with longer timings to complete the task (t (333.1) = -23.4, p < .001). Table-I.

**Table-I:** Comparison of Age, HGS, CST & FEV_1_ time between Control and COPD groups.

	*Group*	*Mean*	*±SD*	*t*	*Sig.*	*95% CI*
Age (years)	Control	41.4	10.1	-1.7	.084	-3.81 to .24
	COPD	43.1	8.2			
BMI (Kg/m^2^)	Control	23.7	3.7	1.9	.064	-.05 to 1.74
	COPD	22.9	5.1			
FEV_1_	Control	95.0	11.4	27.3	< .001*	1.22 to 30.33
	COPD	62.2	10.9			
HGS (kg)	Control	29.6	10.8	6.1	< .001*	4.08 to 8.04
	COPD	23.5	4.6			
CST (sec)	Control	7.6	1.1	-23.4	< .001*	-3.44 to -2.90
	COPD	10.7	1.5			

* Significant Level ≤ 0.05

The difference in age according to gender was not statistically significant (t (126) = 1.8, p = .079). There was significant difference in BMI among gender (24.5 ±2.9 kg/m^2^ *males* vs 23.2 ±4.0 kg/m^2^ *females*) (t (125.8) = 2.2, p = 0.030). Gender comparison of FEV_1_ (98.8 ±4.7 *males* vs 92.3 ±13.7 *females*) showed the difference was significant (t (98.3) = 3.8, p < .001). The HGS in females was significantly less (22.5 ±2.5 kg *females* vs 40.0 ±9.9 kg *males*) on Student’s t-test (t (55.5) = 12.6, p < 0.001). The CST was significantly quick in males (7.0 ±.8 sec *males* vs 7.9 ±1.1 sec *females*) (t (125.6) = -5.5, p < 0.001). Table-II.

**Table-II:** Intra-group comparison of age, HGS, CST & FEV_1_ between gender in Control & COPD groups with Student’s t-test.

*Group*	*Test*	*Gender*	*Mean*	*±SD*	*Sig.*	*95% CI*
Control Group	Age	Male	43.2	9.8	.079	-.37 to 6.73
		Female	40.1	10.1		
	BMI	Male	24.5	2.9	.030*	.13 to 2.55
		Female	23.2	4.0		
	HGS	Male	40.18	9.73	< .001**	14.74 to 20.34
		Female	22.31	2.26		
	CST	Male	7.00	0.81	< .001**	-1.26 to -.59
		Female	7.93	1.11		
	FEV_1_	Male	98.8	4.7	< .001*	1.70 to 3.10
		Female	92.3	13.7		
COPD Group	Age	Male	44.3	7.5	< .001	3.17 to 7.88
		Female	38.8	9.2		
	BMI	Male	23.2	4.9	.074	-.15 to 3.12
		Female	21.7	5.6		
	HGS	Male	25.8	6.4	.002*	-4.70 to -1.33
		Female	22.9	3.8		
	CST	Male	10.7	1.5	.276	-.70 to .20
		Female	10.9	1.6		
	FEV_1_	Male	62.5	11.0	.469	-2.06 to 4.45
		Female	61.3	10.5		

* Significance level P ≤ 0.05

Difference in age (44.3 ±7.5 years *males* vs 38.8 ±9.2 years *females*) was statistically significant (t (254) = 4.6, p < .001). There was no significant difference in BMI among gender (t (80.8) = 1.8, p = .074). No significant difference was observed in FEV_1_ among gender (t (254) = .7, p = .469). Mean HGS in females was significantly less (22.9 ±3.8 kg *females* vs 25.8 ±6.4 kg *males*) on Student’s t-test (t (66.4) = -3.3, p = .002). The difference in CST was not significant between gender (t (254) = -1.1, p = .276). Table-II.

### BMI & FEV_1_ Categories:

Patients were categorized into BMI categories as described in methods. Most common frequency was found in normal BMI groups. Comparison of BMI Categories between Control and COPD groups by χ^2^ test showed no significant differences, details are given in Table-III. Cross-tabulation of GOLD Grade according to Groups showed most common frequency in Control Group of Mild Class (90.6%) and in COPD Group most common frequency was found in Moderate Class (53.6%). Significant difference in frequency was observed by χ^2^ test. Table-IV.

**Table-III:** Cross-tabulation of BMI Categories (Asian) according to Groups with *X*^2^Test

			*Group*		*Total*

			*Control*	*COPD*	
BMI	Underweight	N	7	23	30
Groups	< 18.5	%	5.5%	9.0%	7.8%
	Normal	N	53	102	155
	18.5-22.9	%	41.4%	39.8%	40.4%
	Overweight	N	44	94	138
	23.0-27.5	%	34.4%	36.7%	35.9%
	Obese	N	24	37	61
	> 27.5	%	18.8%	14.5%	15.9%
Total		N	128	256	384
		%	100.0%	100.0%	100.0%

*X*^2^Test = (3, N = 384) = 2.5, p = .471

**Table-IV:** Cross-tabulation of GOLD Grade according to Groups with *X*^2^Test.

			*Group*		*Total*

			*Control*	*COPD*	
GOLD	Mild	N	116	6	122
Grade	≥ 80%	%	90.6%	2.3%	31.8%
	Moderate	N	12	194	206
	50 to <80%	%	9.4%	75.8%	53.6%
	Severe	N	0	56	56
	30 to <50%	%	0.0%	21.9%	14.6%
	Very Severe	N	0	0	0
	<30%	%	0.0%	0.0%	0.0%
Total		N	128	256	384
		%	100.0%	100.0%	100.0%

*X*^2^ Test = (2, N = 384) = 307.5, p < .001

Correlation of Age, BMI, HGS, CST & FEV_1_ was done using Pearson Correlation test and it showed significant correlation between various parameters. Age correlated negatively with HGS & positively with CST, while no correlation was found with BMI & FEV_1_. BMI correlated positively with FEV_1_ and CST but negatively with HGS. HGS correlated positively with FEV_1_ and no correlation was found with CST. No correlation was found of CST with FEV_1_. Table-V.

**Table-V:** Pearson Linear Correlation of Age, BMI, HGS, CST & FEV_1_.

		*Correlations*				

		*Age*	*BMI*	*HGS*	*CST*	*FEV_1_*
Age (years)	Pearson Correlation	1	.034	-.586**	.294**	.097
	Sig. (2-tailed)		.584	.000	.000	.121
BMI	Pearson Correlation	.034	1	-.265**	.205**	.374**
	Sig. (2-tailed)		.584	.000	.001	.000
HGS (kg)	Pearson Correlation	-.586**	-.265**	1	-.059	.179**
	Sig. (2-tailed)		.000	.000	.345	.004
CST (sec)	Pearson Correlation	.294**	.205**	-.059	1	.059
	Sig. (2-tailed)		.000	.001	.345	.349
FEV_1_	Pearson Correlation	.097	.374**	.179**	.059	1
	Sig. (2-tailed)	.121	.000	.004	.349	

**: Correlation is significant at the 0.01 level (2-tailed).

Pearson correlation values with (-) sign means negative correlation

## DISCUSSION

Our study demonstrated important findings that muscle power in COPD Group was less as compared to controls. Muscle weakness plays a pivotal role in the clinical outcome in COPD patients. Muscle weakness is simply the assessment of functionality of muscle and it is assessed by simple and easy tools. Although recently the term sarcopenia has been rejuvenated by Bulow et al., who added muscle strength and physical function like gait speed to the definition of sarcopenia.^13^,^14^ Muscle strength in upper limbs is assessed by hand grip^15^ in COPD patients, while in lower limbs it is assessed by different tools like six minutes’ walk test (6MWT), timed up and go (TUG) test^16^

and chair to stand time (CST). Sarcopenia and muscle weakness in COPD is associated with many disorders like reduced bone mineral density, osteopenia and osteoporosis.^5^ It is shown to be a common finding in stable COPD patients and is also associated with lower exercise tolerance in these patients.^17^ Our findings were consistent with the findings of Miroslav Kovarik et al., where they found that hand grip strength and lower limbs power both were less in COPD patients as compared to healthy control group.^18^ For assessing lower limb function, they utilized 6MWT but in our study, we used CST to assess lower limb function which is equally sensitive.^19^,^20^

We have established that reduced muscle strength was associated with reduced pulmonary function, as we found positive correlation between HGS and FEV_1_. This finding is similar to the study of Han CH et al.^21^ HGS test not only has demonstrated association with mortality in general population,^22^ but also in other disorders including cirrhosis of liver.^23^ It has become an attractive tool for predicting prognosis in COPD, both in clinical practice as well as in research purpose, and is considered to be even better than 6 MWT.^24^ The relationship between HGS and pulmonary function testing showed different results in previous studies as no difference in HGS in COPD patients as compared to non COPD subjects,^25^ while in other studies lower HGS in COPD patients.^26^ These contradictory findings might be due to enrolling COPD patients with different severity, as GOLD Grade 3-4 has significantly lower HGS^26^ than GOLD Grade 1-2. Furthermore, some studies used only GOLD Grade and each GOLD Grade encompass a wide range of FEV_1_. So, we used FEV_1_%, which as a continuous variable is more sensitive when evaluating HGS in relation to COPD severity.

Gender analysis in our study highlighted that in control group males were stronger than females, both in term of HGS and quick CST. Similarly, males in COPD group had good HGS than female COPD patients. This finding was similar to previous studies.^21^,^16^ In our study, in COPD group, females did not show comparative weakness in lower limbs as compared to males, when it comes to CST test. This might be due the fact that in our study, most of the females in COPD group were slightly younger than males. Also, females in COPD group had comparative lower BMI than males in this group. Thus, reproductive age, and lower BMI in females could explain this finding.

Another particularly important finding in our study was that majority of COPD patients had above normal BMI (36.7% overweight and 14.5% obese), with a high mean BMI of 22.9 Kg/m^2^. This contrasts with other studies, where they found that majority of COPD patients were underweight. Effect of BMI on FEV_1_ and clinical outcome of COPD remains controversial. Majority of studies highlighted that lower BMI had been associated with high mortality in COPD patients,^27^ while fewer studies have highlighted that over-weight and obesity is more prevalent in COPD and imposes worse clinical outcomes, low muscle strength in terms of 6MWD and more acute exacerbation of COPD.^28^,^29^ Our study showed positive correlation of BMI with CST and FEV_1_ but negative correlation with HGS in COPD patients. Our finding on BMI were consistent with Lambert AA et al., where he found that 35% of COPD patients were obese, having decrease muscle strength in terms of 6MWT.^29^ This obesity in COPD group in our study might be due to sedentary lifestyle from decreased overall muscle strength. Our study also showed positive correlation of BMI with FEV_1_ meaning patients with low BMI had low FEV_1_ and were in higher GOLD Grade. This effect was also shown by Lim JU et al that Asian BMI classification was more relevant in Asian population.^30^ We also used Asian BMI Classification and found that majority (75.8%) of COPD patients were having moderate airflow limitation (GOLD Grade 2 with a mean post-bronchodilator FEV_1_ was 62.2), followed by severe (21.9%), i.e., GOLD Grade 3.

Main strength of our study is, we used simple and reliable tools to assess muscle strength in COPD patients which can be done easily in clinics. We assessed muscle weakness in both upper as well as lower limbs simultaneously by HGS test and CST test respectively for overall physical functional limitation in COPD patients and compared them with aged matched non COPD group. Whereas most of the previous studies checked muscle function with only single test either HGS or CST or 6MWT. We checked FEV_1_ % as a continuous variable and established its association between muscle weakness and COPD clinical outcome, rather than using only GOLD Grade as a categorical variable, for predicting clinical outcome in COPD patients. In our study correlation between HGS and CST was not statistically significant, reason being the disease may affect differentially upper and lower limbs although both tests individually performed very well in assessment. 31

### Limitations of the study

It is its cross-sectional study design.

## CONCLUSION

We demonstrated muscle weakness in COPD out-patients by simple validated tools. The older COPD patients had less HGS and were slower in doing CST whereas those who had higher FEV_1_ had more HGS. By understanding the magnitude of muscle weakness in COPD patients, future studies can be carried out and focused on strategies to minimize and potentially reverse muscle weakness for better clinical outcome in these patients

### Authors’ Contribution:

**FFZ, BFZ, FSA, NB:** Conception, design, responsible and accountable for the accuracy or integrity of the work.

**FFZ:** Administrative support.

**FSA:** Provision of study materials or patients.

**BFZ, NB:** Collection and assembly of data.

**NB, BFZ, FFZ:** Data analysis and interpretation.

**FFZ, BFZ, FSA, NB:** Manuscript writing.

**FFZ, BFZ, FSA, NB:** Final approval of manuscript.
